# The Batalogue: an overview of betacoronaviruses with future pandemic potential

**DOI:** 10.1093/femsre/fuaf023

**Published:** 2025-05-28

**Authors:** Sarah Baird, Edward C Holmes, Caroline L Ashley, James A Triccas, Megan Steain

**Affiliations:** Sydney Infectious Diseases Institute (Sydney ID), Faculty of Medicine and Health, The University of Sydney, Camperdown, NSW 2006, Australia; School of Medical Sciences, Faculty of Medicine and Health, The University of Sydney, Camperdown, NSW 2006, Australia; Charles Perkins Centre, The University of Sydney, Camperdown, NSW 2006, Australia; Sydney Infectious Diseases Institute (Sydney ID), Faculty of Medicine and Health, The University of Sydney, Camperdown, NSW 2006, Australia; School of Medical Sciences, Faculty of Medicine and Health, The University of Sydney, Camperdown, NSW 2006, Australia; Sydney Infectious Diseases Institute (Sydney ID), Faculty of Medicine and Health, The University of Sydney, Camperdown, NSW 2006, Australia; School of Medical Sciences, Faculty of Medicine and Health, The University of Sydney, Camperdown, NSW 2006, Australia; Charles Perkins Centre, The University of Sydney, Camperdown, NSW 2006, Australia; Sydney Infectious Diseases Institute (Sydney ID), Faculty of Medicine and Health, The University of Sydney, Camperdown, NSW 2006, Australia; School of Medical Sciences, Faculty of Medicine and Health, The University of Sydney, Camperdown, NSW 2006, Australia; Charles Perkins Centre, The University of Sydney, Camperdown, NSW 2006, Australia; Centre for Infection and Immunity, Centenary Institute, The University of Sydney, Camperdown, NSW 2006, Australia; Sydney Infectious Diseases Institute (Sydney ID), Faculty of Medicine and Health, The University of Sydney, Camperdown, NSW 2006, Australia; School of Medical Sciences, Faculty of Medicine and Health, The University of Sydney, Camperdown, NSW 2006, Australia; Charles Perkins Centre, The University of Sydney, Camperdown, NSW 2006, Australia

**Keywords:** coronavirus, zoonoses, pandemic, SARS-CoV-2

## Abstract

The coronavirus disease-19 pandemic has intensified interest in the global diversity of RNA viruses and their ability to jump hosts, with a notable expansion in the number of known betacoronaviruses in wild mammalian species, particularly bats. This has enabled vaccine development research to shift its focus to include a range of severe acute respiratory syndrome coronavirus-1 and severe acute respiratory syndrome coronavirus-2 related viruses from animal species, with the intention of developing broadly protective coronavirus vaccines and therapeutics. However, there is currently a lack of synthesis of this expanding knowledge base of viruses with potential to cause another severe disease outbreak. This has led to many vaccine trials considering protection against a small subset of known betacoronaviruses that poorly approximate the true diversity of this group of viruses. This review aims to synthesize information gained from the recent surge in betacoronavirus characterization, providing a catalogue of viruses exhibiting features that pose a risk to public health, together with a framework for assessing their likelihood of emergence and subsequent transmission through human populations. This information will help inform global pandemic preparedness measures before a novel betacoronavirus outbreak occurs.

## Teaser

A catalogue of betacoronaviruses exhibiting features that pose a risk to public health, together with a framework for assessing their likelihood of emergence and currently identified gaps in immunological protection in the population.

## Lessons from SARS-CoV-1, MERS-CoV, and SARS-CoV-2

The genus *Betacoronavirus* (family *Coronaviridae*) currently comprises five subgenera: *Sarbecovirus, Hibecovirus, Nobecovirus, Merbecovirus*, and *Embecovirus* (Woo et al. 2023). Hibecoviruses and nobecoviruses have not yet been documented in humans, and thus far, members of the subgenus *Embecovirus* largely have been associated with seasonal and mild respiratory disease. However, the past 20 years has seen the emergence of three novel betacoronaviruses from the *Sarbecovirus* and *Merbecovirus* subgenera that are highly pathogenic in humans. Severe acute respiratory syndrome coronavirus-1 (SARS-CoV-1; *Sarbecovirus*) was first detected in Southern China in November 2002, with the resulting outbreak causing an estimated 8098 cases and 774 deaths across 29 countries (Zhao et al. 2003). The first known case of Middle Eastern respiratory syndrome coronavirus (MERS-CoV; *Merbecovirus*) occurred in April 2012 in Jordan (Zaki et al. 2012), and all subsequent outbreaks have been linked with the Arabian Peninsula and emergence events from camels. To date, there have been several MERS-CoV outbreak clusters, totalling 2220 cases and 790 fatalities, equating to a documented mortality rate of ∼35% (Al-Omari et al. 2019). However, population based serological evidence suggests the total number of cases is likely to be underestimated, as MERS-CoV antibodies have been identified in individuals with frequent exposure to camels (Muller et al. 2015). In contrast, the causative agent of the coronavirus disease-19 (COVID-19) pandemic, SARS-CoV-2 (*Sarbecovirus*), formally identified in January 2020 (Wu et al. 2020, Zhou et al. 2020b), has had a far greater global impact, spreading to 114 countries and being declared a global pandemic by the World Health Organisation (WHO) in March 2020. As of May 2025, the COVID-19 pandemic has resulted in over 778 million confirmed cases and 7.09 million deaths (The World Health Organisation 2024). Although SARS-CoV-1, MERS-CoV, and SARS-CoV-2 differ in some aspects of their biology, they share two important features that underlie their severity and warranted urgency in the public health response: a lack of pre-existing immunity and inadequate global preparedness to control their spread and impact.

In the wake of the human and economic cost of the COVID-19 pandemic, the necessity to prepare for the next pandemic pathogen has been asserted by the WHO in their R&D Blueprint for Endemics (The World Health Organisation 2023). The WHO and the Independent Task Force on COVID-19 and other pandemic origins, prevention, and response have highlighted coronaviruses as a family of high pandemic risk (Keusch et al. 2022). Coronaviruses are enveloped, positive-sense, single-stranded RNA viruses, belonging to the order *Nidovirales* (Woo et al. 2023). The family *Coronaviridae* currently comprises four genera: alpha, beta, gamma, and delta. The three major coronavirus outbreaks of the past 20 years have all been caused by members of the genus *Betacoronavirus*—sarbecoviruses and merbecoviruses. In addition, the frequent identification of SARS-CoV-1 and SARS-CoV-2 related betacoronaviruses in bats (particularly microbats of the genus *Rhinolophus*), some of which have the ability to bind to human cellular entry receptors, indicates that another zoonotic spillover and even pandemic event is possible (Menachery et al. 2016, Wacharapluesadee et al. 2021, Zhang et al. 2021). Indeed, a study of people living in rural Myanmar showed that prior to the COVID-19 pandemic, 12% of individuals were seropositive for ‘SARS-like’ viruses, although none had antibodies capable of neutralizing SARS-CoV-2, suggesting spillover of antigenically distinct betacoronaviruses to humans occurs frequently (Evans et al. 2023).

The emergence of SARS-CoV-1, MERS-CoV, and SARS-CoV-2 within the past 20 years likely reflects an increase in these zoonotic spillover events resulting from complex and growing interactions between humans and other animal species (Zinsstag et al. 2018, Plowright et al. 2021). These emergence pathways have been examined in detail in relation to the evolution and origins of SARS-CoV-1, MERS-CoV, and SARS-CoV-2, all of which are postulated to have originated in bat species (Cui et al. 2019, Wong et al. 2019, Boni et al. 2020), jumped into an ‘intermediate’ host and eventually emerged in humans (Wang et al. 2005, Ferguson and Van Kerkhove 2014). This pathway varies slightly in the case of MERS-CoV, which circulated in camels for decades before appearing in humans (Muller et al. 2014, Muller et al. 2015). Understanding the origins and characteristics that allowed these viruses to spillover into humans is of importance for prevention of the next pandemic. As such, this review aims to synthesize the current published knowledge of the distribution and diversity of betacoronaviruses in nature, provide a catalogue of known betacoronaviruses and their key features, and reveal the gaps in individual and population immunological protection against future coronavirus outbreaks.  

## Bats serve as a major reservoir for novel betacoronaviruses

The emergence of a zoonotic virus in humans is dictated by the complex interplay of environmental (wildlife diversity/richness, habitat, and behaviour), anthropologic (proximity of humans to animal reservoirs, such as domestic animals, agriculture, and the wildlife trade), and viral factors (evolution and biogeography) (Forero-Muñoz et al. 2023). Ecological stressors, such as habitat changes, resource availability, disruption to food webs, and species density and overcrowding, can all increase shedding and transmission of pathogens within species and bring wildlife into closer proximity with humans (Plowright et al. 2021, Keusch et al. 2022). Compounding this, agricultural practices and wildlife trade increase contact between wild/domestic animals and humans, and drastically increase the risk of cross-species transmission (Shivaprakash et al. 2021). While not the focus of this review, the significant impact that climate change, modern agricultural practices, and illegal wildlife trade has on these ecological pathways must be considered in first line preventative measures against zoonotic spillover. Prior to the outbreak of SARS-CoV-2, the potential spillover threat of coronaviruses circulating in wild bat species and mammalian wildlife had been highlighted (Menachery et al. 2015, Ge et al. 2016, Menachery et al. 2016).

Bats (order Chiroptera) inhabit all six continents outside the polar regions, represent 20% of all mammalian species, and are a known reservoir of a diverse population of coronaviruses (Teeling et al. 2005, Woo and Lau 2019). Bats exhibit several key biological and physiological characteristics which make them a rich source of viruses primed for spillover to humans. From a behavioural perspective, group co-habitation, capacity for powered flight and the migratory nature of many bat species facilitates the transmission of viruses within and between species, in turn increasing the diversity of the mammalian virome (Carlson et al. 2022). Immunologically, bats have uniquely subdued interferon (IFN), tumour necrosis factor, toll-like receptor, pathogen associated molecular pattern/damage associated molecular pattern and inflammasome signalling (Glennon et al. 2015, Ahn et al. 2016, Xie et al. 2018). The dampening of these anti-viral responses is thought to induce tolerance in bats to RNA viruses including coronaviruses (Zhang et al. 2013, Carlson et al. 2022). This may allow bats to maintain high viral loads during an infection without exhibiting clinical symptoms, which was demonstrated in MERS-CoV infection of Jamaican Fruit Bats (*Artibeus jamaicensis*) (Munster et al. 2016). Together, the high rates of viral co-infection within bats and transmission between individual and different species of bats (Wang et al. 2023), means these mammals present a major risk for the emergence of recombinant novel viruses that may threaten human health.

Although betacoronaviruses have been found in a variety of wildlife species, e.g. members of the subgenus *Embecovirus* commonly found in rodents (Wang et al. 2020b), there is ample evidence that bats, predominantly species from the genera *Rhinolophus* and *Hipposideridae*, harbour a diverse array of betacoronaviruses (Anthony et al. 2017b). Indeed, bat betacoronaviruses have been identifed across the globe, including China (Guan et al. 2003), Japan (Murakami et al. 2020), Cambodia (Delaune et al. 2021), Russia (Alkhovsky et al. 2022), and South America (Caraballo 2022). The risk posed by bat viral reservoirs has been demonstrated by the emergence of SARS-CoV-1 whose origins have been traced back to viruses circulating in wild bat species (Ge et al. 2013, Munster et al. 2016). The evolutionary origins of SARS-CoV-2 are less clear, as a direct progenitor virus (i.e. exhibiting >99% sequence similarity across the genome) has not yet been identified (Voskarides 2022); however, several close relatives (i.e. >96% sequence similarity) have been identified in bats including Banal-52, which was isolated from *Rhinolophus malayanus* (i.e. ‘horseshoe’ bats) in Laos (Temmam et al. 2022). While bats are known to act as a reservoir for coronaviruses, limited contact between humans and bats may constrain direct spillover of viruses, such that virus emergence in humans may require an intermediate species.

## ‘Intermediate’ species serve as reservoirs for transmission of novel betacoronaviruses to humans

Certain animal species can serve as a bridge for viral transmission from wildlife species to humans, and may or may not experience overt disease themselves. These animals typically have direct connection to both wildlife and humans, such as agricultural livestock and those that are traded and trafficked for sale (Lam et al. 2020, Chen et al. 2023, Guo et al. 2023b). Hendra and Nipah virus (*Paramyxoviridae*) circulate in bats, specifically flying foxes (family *Pteropodidae)*, and are known to have spilled over into agricultural animals, including pigs and horses, facilitating transmission to humans (Khusro et al. 2020, Devnath et al. 2022). As with these *Paramyxoviridae* viruses, intermediate species increase opportunities for human exposure to betacoronaviruses (Devnath et al. 2022). Several mammals have been proposed as, or shown to be, the intermediate species for SARS-CoV-1, MERS-CoV, and SARS-CoV-2.  In 2003, a SARS-CoV-1-like virus was isolated from palm civets (*Paguma larvata*) in a Guangdong live animal market, with related viruses also found in raccoon dogs (Guan et al. 2003, Zhao et al. 2005), providing strong evidence these mammals are the species from which SARS-CoV-1 ultimately infected humans (Guan et al. 2003). This was corroborated by a cluster of SARS-CoV-1 cases in 2003/4 linked to a restaurant serving palm civet, where both animals and humans were shown to be infected with SARS-CoV-1 (Wang et al. 2005). While MERS-CoV circulates in dromedary camels, which represent long-term virus reservoirs (Ferguson and Van Kerkhove 2014), it likely has ultimate ancestry in bats (Mohd et al. 2016). The transmission pathway from wildlife to humans has yet to be resolved for SARS-CoV-2. While SARS-CoV-2 related viruses have been found in Malayan pangolins (*Manis javanica)* illegally smuggled into China (Lam et al. 2020, Xiao et al. 2020, Zhang et al. 2020c), they exhibit too many mutational differences to represent viable intermediate hosts and are not the closest relatives of SARS-CoV-2. Similarly, no SARS-CoV-2-like viruses have yet been identified in civets or racoon dogs. Identification of an intermediate species for SARS-CoV-2 would help to elucidate the evolutionary origins of the virus, direct surveillance and assist prevention measures for future zoonoses.

## Defining the pandemic potential of emergent betacoronaviruses

**Figure 1.**
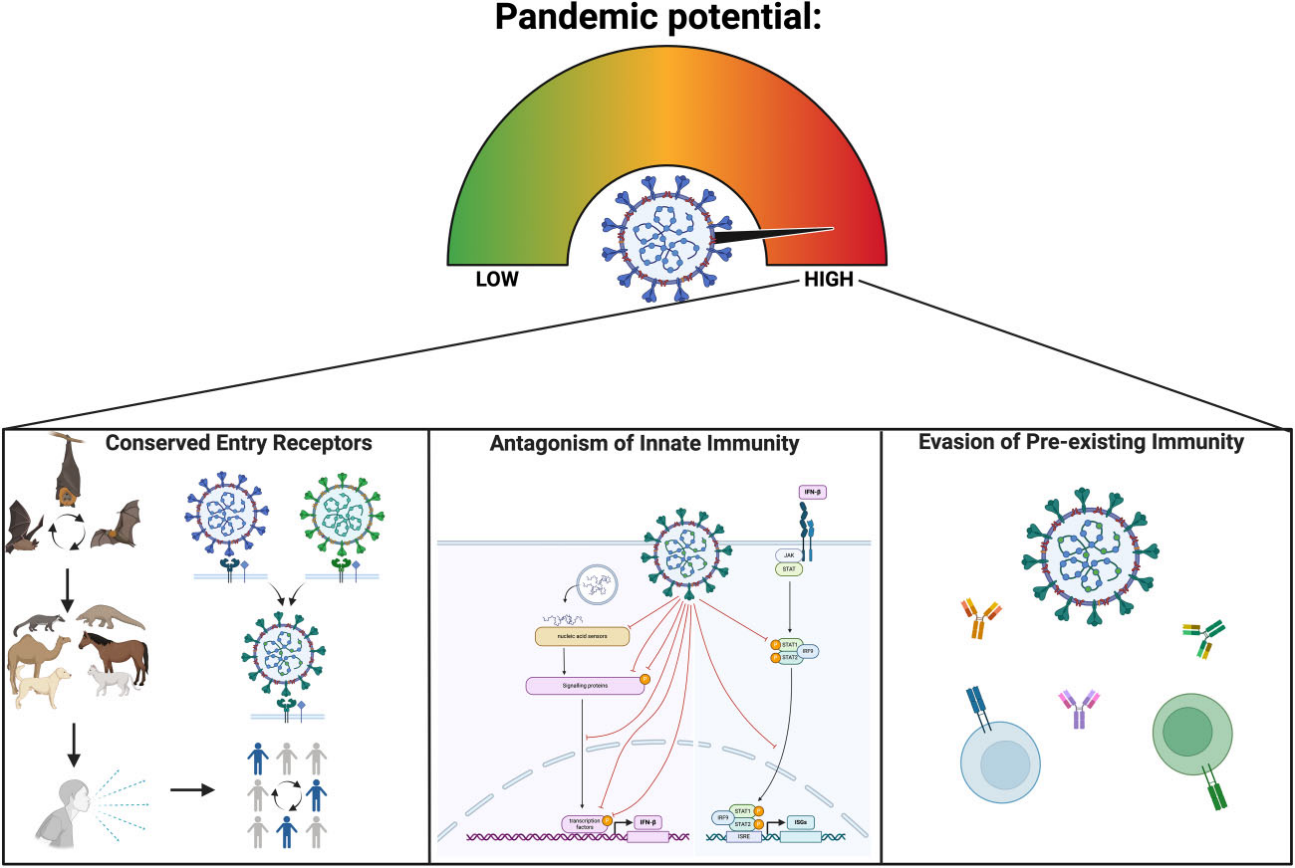
Framework for defining the pandemic potential of betacoronaviruses. Created in BioRender. Ashley, C. (2025) https://BioRender.com/n2ekii0

Given the availability of a possible intermediate host and the opportunity to spillover into humans, the pandemic potential of a novel betacoronavirus should be assessed by several factors, including the ability to; (i) bind to human entry receptors and other mammalian orthologues of these proteins, (ii) hamper innate anti-viral immune responses, and (iii) evade pre-existing population immunity induced by infection or vaccination against related viruses (Fig. 1) (Warren and Sawyer 2023). The SARS-CoV-2 virus first sequenced in patients in Wuhan, China, in early 2020 (Zhou et al. 2020b), exhibited these characteristics. The virus could efficiently bind to human and mammalian orthologues of angiotensin converting enzyme 2 (ACE2), dampen anti-viral, and pro-inflammatory signalling (Yao et al. 2023), and was poorly cross-neutralized by convalescent sera from individuals following SARS-CoV-1 or common cold coronavirus infections (Zhao et al. 2003). Some of these characteristics were likely acquired through intra-sarbecovirus recombination events during its evolutionary history (Li et al. 2020a). Indeed, recombination is commonplace in coronaviruses, and recombination within the spike protein has been proposed to generate genomic configurations that by chance facilitate cross-species spillovers (Wells et al. 2023). Thus, recombination, the ability to mutate and evolve rapidly, along with the capacity bind to conserved cellular receptors are major contributors to coronavirus epidemic/pandemic potential.

### Features of concern in SARS-CoV-1, SARS-CoV-2, and MERS-CoV

One of the primary factors permitting the spillover of SARS-CoV-1, SARS-CoV-2, and MERS-CoV is their capacity to bind human and mammalian orthologues of ACE2 (SARS-CoV-1/-2) and Dipeptidyl peptidase 4 (DPP4; MERS-CoV). Receptor binding models have been used to predict host range, tissue tropism, and binding affinity of the SARS-CoV-1/SARS-CoV-2 spike protein and human ACE2 to assess the key interacting residues. Several residues in spike, including 442, 472, 479, 480, and 487 in SARS-CoV-1 or 445, 486, 494, 493, and 501 in SARS-CoV-2, have been shown to largely dictate host range and tissue tropism (Wan et al. 2020); however, many more residues are known to interact with ACE2 (Lan et al. 2020). SARS-CoV-1 was pre-adapted to both civet ACE2 and human ACE2, which provides a structural basis for its ability to jump the species barrier into and from the intermediate species, civets, into humans. This is similarly seen for MERS-CoV which can bind to human and camel DDP4, and can be transmitted within and between both species (Drosten et al. 2014, Memish et al. 2014). Several studies during the SARS-CoV-2 pandemic investigated the broad host range of the virus, which includes bat species, wildlife such as pangolins and racoon dogs, domestic animals, including cats and livestock, and zoo animals such as lions and tigers (Bouricha et al. 2020). Thus, receptor promiscuity is of particular concern for cross-species transmission of pathogenic coronaviruses. Receptor binding affinity is also associated with intra-species transmission, e.g. while SARS-CoV-1 was not as efficiently transmitted between individuals, SARS-CoV-2 was able to bind with higher affinity to human ACE2 through the asparagine at position 501, leucine at 455 and phenylalanine at 486 (Wan et al. 2020). This greater affinity of SARS-CoV-2 spike to human ACE2 may account, in part, for its increased transmissibility compared to SARS-CoV-1. Additionally, an insertion point at the S1/S2’ junction, representing a polybasic furin-like protease cleavage site, is present in SARS-CoV-2 and MERS-CoV (albeit with different sequences), but is absent in SARS-CoV-1. This site mediates cleavage of the spike S1 and S2 subunits during biosynthesis. In addition to higher affinity binding to ACE2, this polybasic furin-like protease cleavage site has been proposed to significantly increase the infectivity of SARS-CoV-2 compared to SARS-CoV-1 (Zhou et al. 2020a, Peacock et al. 2021.

While these features initially endowed significant survival advantage to these novel viruses, the mutations that arose over the course of the SARS-CoV-2 pandemic continuously challenged pandemic control measures and would likely pose a similar difficulty in future pandemics caused by betacoronaviruses.  The first of these was the D614G mutation that likely emerged early in the Wuhan outbreak and, based on the GISAID SARS-CoV-2 sequence database, was present in only 10% of sequenced virus prior to 1st March 2020. Yet, by May 2020, this had become the globally dominant variant (78% of sequences) (Korber et al. 2020, Zhang et al. 2020a), and it is present in all subsequent variants of interest and concern. This aspartic acid to glycine substitution in the C-terminal domain of S1 results in three-fold higher viral loads based on reverse transcription-polymerase chain reaction (RT-PCR) threshold values from human samples, and 2.3–9 fold higher viral titres in lentivirus pseudotyped models (Korber et al. 2020). The emergence of the D614G mutant occurred in parallel with reports of the doubling time of SARS-CoV-2 infections decreasing from 6 to 3 days (Pellis et al. 2021). The D614G mutation stabilizes the receptor binding domain (RBD) into an up conformation required for access to ACE2, which may explain the molecular basis of its increased infectivity (Jackson et al. 2021) and why it served as ‘gate keeper’ mutation for the adaptation of SARS-CoV-2 to humans. 

The Omicron variant and its sub-lineages are also of particular interest as their substantial antigenic variation profoundly impacted the efficacy of currently available SARS-CoV-2 vaccines and monoclonal antibodies (mAb) (Huang et al. 2022). These lineages carry several common mutations to the Beta and Delta variants including N501Y, which further increases ACE2 binding affinity, and E484K (E484A in Omicron/sublineages) which allows evasion of neutralizing antibody responses (Chatterjee et al. 2023, Joshi et al. 2021). Furthermore, while the Beta variant had 21 mutations, 9 located in spike, the BA Omicron sublineage accumulated 50 amino acid mutations in its genome, with 30 located in the spike protein and half of these in the RBD (Cui et al. 2022). The development of these evolutionarily advantageous genetic mutations is an important consideration in a future spillover of a pandemic virus from wildlife.

## Emergent Betacoronaviruses with pandemic potential

### Sarbecoviruses

**Figure 2.**
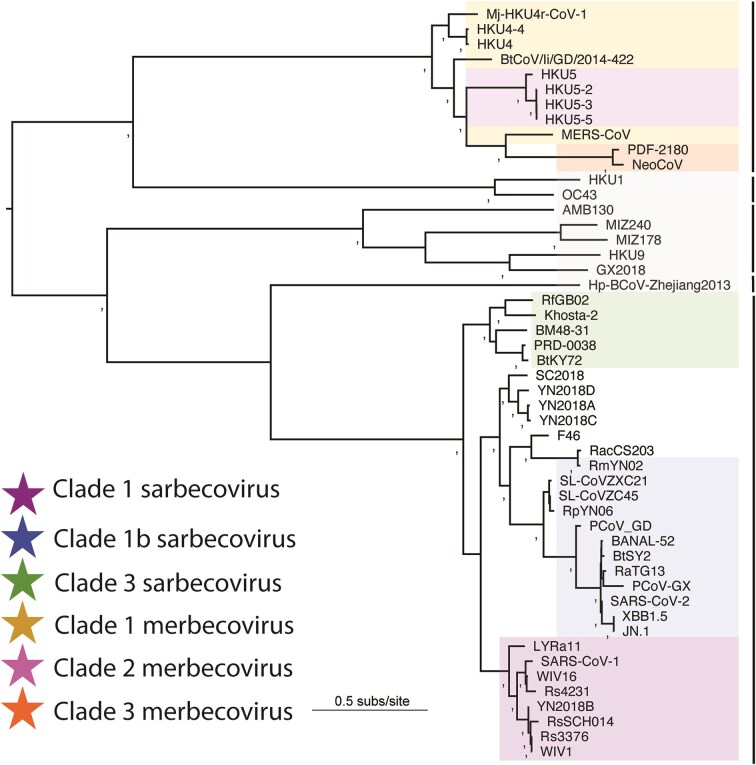
Evolutionary relationships among the betacoronavirus based on a phylogenetic analysis of full-length spike sequences. The amino acid sequences were aligned in CLC workbench with a phylogenetic tree then estimated using the maximum likelihood method available in PhyML (Guindon et al. 2010), employing the LG + G4 model of amino acid substitution. SH-like branch support values >90% are marked by an * at the relevant nodes. The tree is mid-point rooted for clarity only and all horizontal branch lengths are scaled according to the number of amino acid substitutions per site.

**Figure 3.**
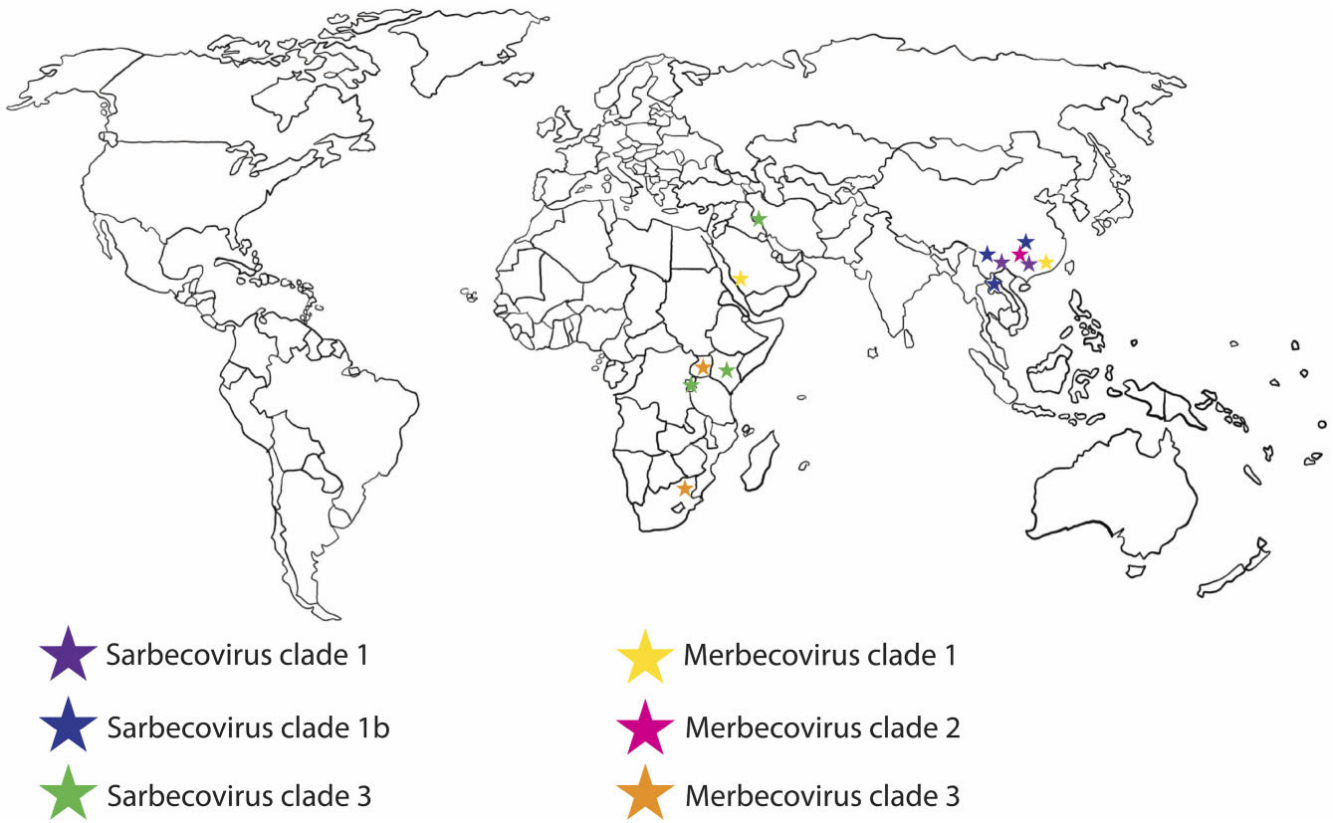
Global geographic distribution of known betacoronaviruses in bats and humans. Viral subgenus and clades are colour coded as shown in legend.

There are three clades of sarbecoviruses that are of potential significance to public health: (i) the SARS-CoV-1 related (SARSr) or SARS-like (SL-CoVs) coronaviruses -clade 1, (ii) SARS-CoV-2 related (SARS-2r)- clade 1b, and (iii) the European/African clade 3 (Lee et al. 2023)(Fig. 2). Clade 2 sarbecoviruses contain 3 deletions in the RBD that negate binding to human ACE2 with sufficient affinity to pose a high risk of spillover (Letko et al. 2020). The classification of sarbecoviruses within these clades can vary depending on the region sequenced, i.e. the RNA-dependent RNA-polymerase (RdRp), Spike, RBD, or full-length genome (Wang et al. 2023). Interestingly, clades 1 and 1b, while genomically distinct, partially overlap in their geographic distribution, particularly around Yunnan province, China (Fig. 3). While clade 1 viruses are more common in Yunnan and further north in China, clade 1b viruses are generally found further south around Laos, Cambodia, Thailand, Vietnam, Myanmar, and Malaysia (Holmes 2024). However, antibodies reactive to SARS-CoV-1 antigens have been found in species of flying foxes *(Pteropus)* in Adelaide, Australia (Boardman et al. 2021) and Christmas Island in the Indian Ocean (Pulscher et al. 2022). At face value, these data suggest that the geographic and species classifications are a limited reflection of the true distribution of these viruses. The clade 3 viruses span a much larger region, including Russia and East Africa (Fig. 3).  While this geographic distribution is useful to contextualize the large number of known sarbecoviruses, ACE2 usage is the primary determinant of spillover risk for sarbecoviruses. Binding affinity and ACE2-RBD complex stability largely dictate the preferential receptor usage of these viruses (Wang et al. 2020a, Chakraborty et al. 2022).

### Clade 1 sarbecoviruses

Clade 1 comprises viruses that can bind to human ACE2, including SARS-CoV-1, SL-CoV-WIV1, WIV16, Rs3376, RsSCH014, SL-CoV-LS1, and LYRa11 (Table 1). As with SARS-CoV-1, the S1/S2’ polybasic furin-cleavage site has not been discovered in any viruses belonging to this clade to date. As such, the capacity of these viruses to bind with high affinity to ACE2, as well as the presence of any cross-neutralizing antibodies in human sera post-SARS-CoV-2 infection and/or vaccination, is of primary significance to their potential pathogenesis (Li et al. 2005).

**Table 1.** Overview of key betacoronaviruses with known or potential capacity for human infection.

Virus	Region found	Human receptor usage	Neutralization by human serum-post SARS-CoV-2 infection and/or vaccination	Novel features	References	Accession numbers (spike locus)
Sarbecoviruses						
Clade 1						
SARS-CoV-1 (Tor2)	First detected in Guangdong, China	ACE2	Limited cross-neutralization by mRNA vaccinated patient sera	First significant Sarbecovirus outbreak	(Peiris et al. 2003, Garcia-Beltran et al. 2021)	NC_004 718 (YP_009825051.1)
WIV-1	Yunnan, China	ACE2	Not neutralized by SARS-CoV-2 vaccine sera	Virus isolated; ORFX present, modulates NF-kB responses	(Ge et al. 2013, Garcia-Beltran et al. 2021)	KF367457.1 (AGZ48828.1)
WIV-16	Yunnan, China	ACE2		Closest known relative of SARS-CoV-1; ORFX present	(Yang et al. 2015a)	KT444582 (ALK02457.1)
RsSCH014	Yunnan, China	ACE2	Limited cross-neutralization with SARS-CoV-2 vaccine and/or vaccination	0/5 ACE2 binding residues from SARS-CoV-1 conserved	(Ge et al. 2013, Tan et al. 2021)	KC881005.1 (AGZ48806)
Rs3376	Yunnan, China	Suggested to be ACE2		2/5 ACE2 binding residues from SARS-CoV-1 conserved	(Ge et al. 2013)	KC881006.1 (AGZ48818)
LYRa11	Yunnan, China	ACE2		ORF4 absent	(He et al. 2014)	KF569996.1 (AHX37558)
Rs4231	Yunnan, China	ACE2			(Hu et al. 2017)	KY417146 (KY417146.1)
SL-CoV-LS1	Yunnan, China	ACE2			(Wang et al. 2023)	*NA*
Clade 1b						
Ancestral SARS-CoV-2	First detected in Wuhan, China	ACE2		Binds hACE2 with 4-fold higher affinity than SARS-CoV-1	(Wu et al. 2020, Zhou et al. 2020b)	YP_009724390.1
RaTG13	Yunnan, China	ACE2	Well neutralized by SARS-CoV-2 vaccine sera	Binds many ACE2 orthologs	(Ge et al. 2016, Zhou et al. 2020b, Cantoni et al. 2022)	MN996532.2 (QHR63300)
Banal-52	Laos	ACE2	Well neutralized by SARS-CoV-2 vaccine sera	Closest relative of SARS-CoV-2 to date	(Temmam et al. 2022)	MZ937000 (UAY13217)
RmYN02	Yunnan, China	ACE2		Contains PRR S1/S2’ insertion point	(Zhou et al. 2020a)	EPI_ISL_412 977 (GSIAD)
RpYN06	Yunnan, China	ACE2		Highly similar in genetic identity to SARS-CoV-2 in all gene regions other than spike	(Zhou et al. 2021)	MZ081381 (QWN56252.1)
PCoV-GD	Pangolins rescued from illegal trafficking	ACE2	Highly neutralized by SARS-CoV-2 vaccinated sera	Binds hACE2 with higher affinity than SARS-CoV-2	(Xiao et al. 2020, Nie et al. 2021)	EPI_ISL_410 721
PCoV-GX	Pangolins rescued from illegal trafficking	ACE2	Mildly neutralized by SARS-CoV-2 vaccinated sera		(Lam et al. 2020, Nie et al. 2021)	CoV_EPI_ISL_410 542
bat SL-CoV-CX1/BtSY2	Yunnan province, China	Suggested to be ACE2		Evidence of recombination between clades 1 and 1b	(Wang et al. 2023)	*OQ934005*
SL-CoV ZXC21	Zhejiang province, China	Suggested to be ACE2			(Hu et al. 2018)	MG772934
SL-CoVZC45	Zhejiang province, China	Suggested to be ACE2			(Hu et al. 2018)	MG772933
Clade 3						
Khosta-2	Sochi, Russia	ACE2	Poorly neutralized by SARS-CoV-2 vaccine sera		(Alkhovsky et al. 2022, Seifert et al. 2022)	MZ190138.1 (QVN46569)
BtKY72	Kenya, East Africa	ACE2		Requires exogenous protease treatment for entry into hACE2 cells	(Tao and Tong 2019)	KY352407.1 (APO40579.1)
PDF-0038	Rwanda	ACE2			(Wells et al. 2021)	MT726045 (QTJ30153.1)
**Merbecoviruses**						
Clade 1						
Mers-CoV		DPP4			(Zaki et al. 2012)	NC_019 843 (YP_009047204.1)
HKU4	Guangdong Province, China	DPP4		Bat adapted	(Woo et al. 2006)	NC_009 019 (YP_001 039 953)
MjHKU4-CoV-1	Pangolins rescued from illegal trafficking	DPP4		Contains S1/S2’ furin-like cleavage site and can bind human, pangolin and bat orthologues of DPP4	(Chen et al. 2023)	CRA006906
BtCoV/Ii/GD/2014–422	Guangdong Province, China	DPP4	No neutralization by MERS-CoV antisera		(Luo et al. 2018, Tse et al. 2023)	MG021452 (AVV62537.1)
Clade 2						
HKU-5	Guangdong Province, China	ACE2			(Woo et al. 2006, Catanzaro et al. 2024, Chen et al. 2025, Park et al. 2025)	
Clade 3						
NeoCoV	South Africa	ACE2	No neutralization by SARS-CoV-2 vaccinated sera	ACE2 binding relative of MERS-CoV	(Ithete et al. 2013, Xiong et al. 2022)	KC869678 (AGY29650.2)
PDF-2180	Uganda	ACE2	No neutralization by SARS-CoV-2 vaccinated sera	ACE2 binding relative of MERS-CoV	(Anthony et al. 2017a, Xiong et al. 2022)	NC_034440.1 (YP_009361857.1)

Discovered in China in 2013, WIV1 was the first SARS-like virus to be successfully isolated, as opposed to sequenced, from bats (Ge et al. 2013). It shares 95% sequence identity in the RBD to SARS-CoV-1 and contains three point mutations in the RBD-ACE2 interacting residues (442S, 472F, and 487N), yet can still infect humans cells via ACE2 (Chakraborty et al. 2022).

RsSCH014 and Rs3376 were sequenced in the same study that isolated WIV1 (Ge et al. 2013). The full genome of Rs3367 is 99.9% and the RBD is 100% similar in sequence to WIV1. For this reason, many of the similarities between WIV1 and SARS-CoV-1 extend to Rs3367. RsSCH014 shares ∼95% whole sequence identity with SARS-CoV-1, while the RBD of RsSCH014 has considerably lower amino acid identity to SARS-CoV-1 compared to WIV-1. Despite these differences, RsSCH014 has been shown to bind human ACE2 (hACE2) with comparable affinity to SARS-CoV-1 in a VSV-G pseudotyped model (Letko et al. 2020).

WIV16 was sequenced, also from China, in 2015 and is the closest known relative of SARS-CoV-1 across the full-genome (Yang et al. 2015a), with 95% amino acid homology in the RBD. The genome organization of WIV1 and WIV16 is almost identical, with both harbouring an additional novel ORF (ORFX) between ORF7 and 8, that is not present in any other currently identified SARS-like viruses. It was speculated that the RBD similarities between WIV1 and WIV16 are evidence of a recombination event having occurred between WIV1 and a SARS-CoV-1 ancestor, producing WIV16’s spike protein (Yang et al. 2015a).

LyRa11 was identified in 2014 in *R. affinis* bats from Yunnan province, China. The virus has a full-genome sequence identity of 91% to SARS-CoV-1, although ORF4 is absent (He et al. 2014). LyRa11 shows 92.5% amino acid identity with the SARS-CoV-1 RBD, and 2/5 conserved key hACE2 interacting residues, N479N and Y491Y (He et al. 2014, Wang et al. 2017). In a VSV-G pseudotyped model, LyRa11 enteres hACE2 expressing cell lines, albeit to a lower degree than SARS-CoV-1, WIV1, Rs3367, and RsSCH014 (Letko et al. 2020).

Finally, LS1 was identified in two different species of bats in the Yunnan province, China (Wang et al. 2023), and displays high genetic identity (93%) to SARS-CoV-1 across the whole genome. However, while it has 98.13% similarity in the N-terminal domain of the spike protein, this decreases to 88.61% in the RBD. Its affinity for hACE2 has not been investigated.

### Clade 1b sarbecoviruses

The clade 1b viruses include SARS-CoV-2 and its bat relatives, such as bat-SL-CoVZC45, bat-SL-CoVZXC21, RaTG13, RmYN02, BANAL-52 (and related viruses from Laos), bat SL-CoV-CX1, and the pangolin viruses PCoV-GD and PCoV-GX. While several other viruses have been sequenced in this clade, they are yet to be characterized (Han et al. 2019), or don’t bind to ACE such as RacCS203 (Wacharapluesadee et al. 2021). RaTG13 was initially discovered in *R. affinis* bats in a mineshaft located in Yunnan province in 2013, yet only a partial sequence was obtained (Ge et al. 2016, Zhou et al. 2020b), with the complete genome not published until early 2020 (Zhou et al. 2020b). RaTG13 shares 96.2% nucleotide identity across the whole-genome with SARS-CoV-2, 92.9% in the S gene, but only 89.3% in the RBD (Boni et al. 2020, Zhou et al. 2020b, Liu et al. 2021). The mutations exhibited by RaTG13 in the RBD result in decreased binding affinity to hACE2 compared to SARS-CoV-2. This has been validated *in vitro*, where incorporation of two single point mutations D501N and H505Y, which are present in the SARS-CoV-2 RBD, increase infectivity of RaTG13 to hACE2 and bat ACE2 by 100-fold (Li et al. 2023).  RaTG13 also exhibited binding affinity to ACE2 orthologs from other animals, including but not limited to primate, bat, rabbit, horse, pig, and cat (Liu et al. 2021).

Bat-SL-CoVZC45 and bat-SL-CoVZXC21 were sampled from *R. sinicus* bats from Zhejiang province, China in 2017 and 2015, respectively (Hu et al. 2018). At the time of their discovery, phylogenetic analysis of available sarbecovirus spike nucleotide sequences designated these two bat viruses as a new clade, distinct from SARS-CoV-1 (Hu et al. 2018). SL-CoVZC45 and SL-CoVZXC21 shared 97% genomic sequence identity to each other and relatively low identity (81%) across the whole genome to SARS-CoV-1. As suggested by their large phylogenentic distance in the spike gene to SARS-CoV-2 (∼75%) (Lu et al. 2020), these viruses have been shown to bind hACE2 with low affinity (Letko et al. 2020).

BANAL-52, a virus sampled from *R*. *malayanus* bats in Laos in 2022 (Temmam et al. 2022), is the closest currently identified relative of SARS-CoV-2. BANAL-52 is 96.8% similar across the whole genome and 97.4% similar in the RBD, which shares all the key amino acid residues (Ou et al. 2023). BANAL-52 binds with slightly higher affinity to human ACE2 in cell line models than SARS-CoV-2 (Ou et al. 2023), but lacks a furin cleavage site at the S1/S2 junction. The insertion of an exogenous S1/S2 cleavage site in BANAL-52 increased pseudovirus transduction efficiency ∼2-fold in human epithelial CaLu3 cells (Ou et al. 2023). As such, the presence of this cleavage site in a bat SARS-CoV-1/2 related virus would be of particular concern. 

Prior to the discovery of BANAL-52, RpYN06, also sampled from *Rhinolophus* bats in Yunnan, was one of the closest relatives of SARS-CoV-2 across the full genome, at 94.5% genetic similarity. However, the relatively large dissimilarity in the spike gene of RpYN06 at 76.3% indicates that while this virus may share a backbone with SARS-CoV-2, the spike gene may have been acquired from elsewhere, and as such binds to bat but not human ACE2 (Zhou et al. 2021).

RmYN02 was similarly isolated from *R. malayanus* bats sampled in Yunnan in 2019. RmYN02 shares 93.3% sequence identity across the whole genome to SARS-CoV-2, 71.9% in the S gene, but exhibits much lower in similarity (61.3%) in the RBD to SARS-CoV-2 than RaTG13 (Zhou et al. 2020a). However, in the ORF1ab gene, RmYN02 is 97.2% similar to SARS-CoV-2. As with RpYN06, these differences in genomic region similarity to SARS-CoV-2 are indicative of past recombination events. A defining feature of interest is the presence of a three amino acid insertion (PAA) at the S1/S2 junction (Zhou et al. 2020a). Although this insertion region does not match that of the four amino acid furin cleavage site (PRRA) in SARS-CoV-2, it demonstrates that mutations may ultimately give rise to this pathogenic characteristic in nature (Zhou et al. 2020a). Whether RmYN02 can bind to hACE2 is yet to be tested. It was hypothesized that the absence of a disulfide bond in the external subdomain of the RBD in RmYN02, which is conserved across SARS-CoV-1, SARS-CoV-2, RaTG13, and PCoV-GX/GD, may substantially reduce its binding affinity to hACE2 or ablate it all together (Zhou et al. 2020a).

In 2019, two viruses, PCoV-GX (Lam et al. 2020) and PCoV-GD (Xiao et al. 2020), were isolated from pangolins rescued from illegal wildlife trade into China. PCoV-GX (from animals brought into Guangxi province) shares 85.5% sequence identity with SARS-CoV-2, while PCoV-GD (from Guangdong province) shares 92.4%. Of particular note, the RBD of PCoV-GD is 97.4% homologous in amino acid identity to SARS-CoV-2 (Xiao et al. 2020). However, PCoV-GD binds to hACE2 with higher affinity than SARS-CoV-2, which suggests this virus may be a significant threat for spillover into humans (Nie et al. 2021). While the RBD of PCoV-GX has a considerably lower amino acid sequence identity to that of SARS-CoV-2 (86.7%), it also binds to hACE2 with higher affinity than SARS-CoV-2, albeit less so than PCoV-GD (Nie et al. 2021, Zhang et al. 2021). As with RaTG13 and RmYN02, both these pangolin viruses bind to several domestic and wild animal orthologues of ACE2 (Li et al. 2020b). The presence of these SARS-CoV-2-like betacoronaviruses in pangolins which are capable of binding human, pangolin and bat ACE2, demonstrates their adaptability to mammalian hosts. This characteristic poses a substantial concern for future spillover events.

Finally, Bat SARS-like virus CX1/BtSY2, isolated in 2022 in *R. marshalli* and *R. pusillus* bats in Yunnan, China, shares 92% genetic identity to SARS-CoV-2 (Wang et al. 2023). While this virus clusters with the SARS-2r viruses based on the RBD and NTD of spike, the RdRp sequence places it within the SARS-1r virus clade. Of particular note, it’s mosaic genome is indicative of a recombination event between the clade 1 and 1b viruses. Although CX1/BtSY2 shares 93.7% genome identity to SARS-CoV-2 in the RBD (Temmam et al. 2022), whether CX1/BtSY2 can efficiently bind ACE2 for cell entry has yet to be established. However, as there are only 5 amino acid substitutions in the RBD (A372T, F486L, Q489H, N501Y, and H519N) compared to ancestral SARS-CoV-2, it is likely this virus does share this entry receptor with the others in its clade (Wang et al. 2023). Interestingly, the N501Y mutation present in the CX1/BtSY2 RBD also occurs in the SARS-CoV-2 RBD in all viral variants from Alpha to Omicron, and was reported to increase affinity for hACE2 (Joshi et al. 2021, Liu et al. 2022).

### Clade 3 sarbecoviruses

Clade 3 sarbecoviruses span a large geographical distance, from Europe and Russia to Africa and include BtKY72, Khosta-2, and PRD-0038. These viruses contain one deletion in the RBD relative to SARS-CoV-2 which impairs their ability to bind hACE2 with high affinity, as well as missing ORF8 which is present in all other bat SL-CoVs to date (Seifert et al. 2022). Khosta-2 was discovered in *Rhinolophus and Hipposideros* bats (Alkhovsky et al. 2022). The RBD of Khosta-2 shares 69% amino acid identity with the putative ancestral SARS-CoV-2. While it was originally hypothesized that the truncated variable loop of Khosta-2 would prevent hACE2 binding, the virus was shown to infect hACE2 expressing cell lines in a pseudotyped virus model to a similar degree as RaTG13 (Seifert et al. 2022, Starr et al. 2022). Despite the substantial genetic distance between Khosta-2 and SARS-CoV-1/2 its ability to bind human ACE2 highlights Khosta-2 as a virus of considerable risk to humans.

Two other notable clade 3 viruses, BtKY72 and PRD-0038, were discovered in Kenya in 2017 (Tao and Tong 2019) and Rwanda in 2021 (Wells et al. 2021), respectively. While these viruses do not bind to hACE2 with notable affinity, a single amino acid substitution in their RBD (T487W) can broaden their receptor tropism and allow entry into hACE2 expressing cell lines (Starr et al. 2022, Lee et al. 2023). Exogenous protease treatment has also been shown to enhance entry into hACE2 cell lines in pseudotyped VSV-G BtKY72-RBD model.

### Merbecoviruses  

MERS-CoV shares a similar genome structure to other betacoronaviruses (Wang et al. 2016). However, while the core RBD of MERS-CoV spike is structurally equivalent to SARS-CoV-1, the external domain is highly divergent, contributing to the differential receptor usage, as the virus utilizes DPP4 rather than ACE2. It was originally hypothesized that the human-to-human transmissibility of merbecoviruses would be limited due to their use of DPP4, which in humans is predominantly expressed in the lower respiratory tract, unlike hACE2, which is expressed in both the upper and lower respiratory tracts (Widagdo et al. 2019). However, this theory was challenged by the discovery of ACE2-binding merbecoviruses. Receptor usage can delineate the merbecovirus clades, with so-called clade 1 merbecoviruses comprising those that bind human, bat, camel and/or pangolin DPP4, including MERS-CoV, HKU4, Bt-CoV-422, and Mj-HKU4r-CoV-1. The HKU5 viruses, including HKU5-2, -3, and -4, together with BtVs-betaCoV/SC2013, form clade 2 and bind to ACE2 in a species-specific manner, with affinity predominantly for bat orthologues (Catanzaro et al. 2024, Park et al. 2025). NeoCoV and PDF-2180, discovered in Africa, also utilize ACE2 and are the only known viruses to form merbecovirus clade 3. Finally, clade 4 includes the hedgehog virus Erin-CoV, for which the entry receptor is unknown (Catanzaro et al. 2024). In addition to these receptor specificities, the MERS-CoV spike contains a furin cleavage site at the S1/S2 junction, in a similar manner to SARS-CoV-2 but with a different sequence motif, and this has also been shown to be critical for entry into human DPP4 expressing cell lines (Millet and Whittaker 2014).

### Merbecovirus Clade 1

HKU4 is a bat virus discovered in Guangdong Province, Southern China in 2006 (Woo et al. 2006) that utilizes DPP4 but has very low affinity to the human orthologue (Wang et al. 2014). Variations between MERS-CoV and HKU4 in the furin cleavage site and endosomal cystine cleavage site have been shown to dictate the infection capacity of MERS-CoV into human DPP4 expressing cells, a property not possessed by HKU4 (Yang et al. 2015b).  Insertion of S746R and N762A point mutations into the HKU4 S1/S2 cleavage site enables the virus to efficiently enter human cell lines (Wang et al. 2023). While HKU4 can be considered low risk to humans, the presence of a similar cleavage site in a closely related virus discovered in pangolins, MjHKU4r-CoV-1, indicates that it is possible for merbecoviruses to acquire such virulence characteristics when the species barrier is crossed (Guo et al. 2023b). MjHKU4r-CoV-1 was identified in a surveillance study of Malayan pangolins, in animals seized from mammals trafficked out of Southeast Asia (Chen et al. 2023). This virus is 69.7% similar to MERS-CoV and 86.6% similar to HKU4. Of note, MjHKU4r-CoV-1 utilizes bat, pangolin, and human DPP4 *in vitro*, and 12.8% of the 86 pangolins seized from trafficking were seropositive for MjHKU4r-CoV-1, indicative of extensive circulation in pangolins (Chen et al. 2023). Concerningly, the unique furin cleavage site in this virus (RQQR) enables proteolytic cleavage by human furin proteases. Like MERS-CoV, MjHKU4r-CoV-1 antagonizes type 1 IFN signaling (Chen et al. 2023). As outlined previously, this ability to supress host innate immune responses is a key determinant of pandemic potential and disease pathogenesis. 

BtCoV/Ii/GD/2014–422 and BtCoV/Ii/GD/2013–845 are viruses discovered in la io *(Vespertilionidae)* bats in Guangdong province, China (Luo et al. 2018). These viruses are ∼75% homologous in amino acid identity to MERS-CoV across the whole genome, but this decreases to 64.7–64.9% in spike, and 61.9–63.6% in the RBD. Interestingly, the RBD of these viruses were more similar to HKU4 than MERS-CoV, suggesting a recombination event between an ancestor virus and HKU4 in their evolution. BtCoV-422 binds human and bat DPP4, and entry into hDPP4/bDPP4 cell lines was not inhibited by MERS-CoV mAbs or antiserum (Luo et al. 2018). Several genetically similar MERS-CoV related viruses have been identified in *Vespertilionidae* bats, including in Italy (Lelli et al. 2013) and Spain (Falcón et al. 2011); however, no study to date has characterized their entry receptor usage.

### Merbecovirus Clade 2

Clade 2 Bat-CoV HKU5 viruses were first identified in Japanese pipistrelles (*Pipistrellus abramus*) from Hong Kong (Woo et al. 2006). Initial studies using a full-length infectious clone showed that trypsin treatment facilitated infection of Vero cells. These viruses use ACE2 for entry, with a clear preference for *P. abramus* ACE2 (Catanzaro et al. 2024, Park et al. 2025). A recent study showed low level HKU5 replication in human Caco-2 cells in the presence of trypsin (Catanzaro et al. 2024), and a single amino acid substitution in the RBD of HKU5-21s was shown to increase entry into hACE2 expressing cells (Catanzaro et al. 2024). More recently, HKU5-2 isolates were shown to infect human cell lines expressing ACE2 efficiently, and represent a significant spill-over threat (Chen et al. 2025). Additionally, HKU5-like viruses have also been found in the lungs of farmed mink in China suffering from pneumonia. Mink could therefore plausibly act an intermediate species to facilitate a spillover of these viruses into humans (Zhao et al. 2024). Given that HKU5 viruses are antigenically distinct to MERS-CoV, and like all betacoronviruses can adapt to sub-optimal receptor interactions, it is imperative that these viruses are considered when designing pandemic countermeasures.

### Merbecovirus Clade 3

NeoCoV and its sister virus, PDF-2180, represent an interesting case for substantial genetic diversity and wide geographic distribution within clades of viruses. NeoCoV was discovered in 2013 in *Laephotis capensis* bats, in South Africa, and PDF-2180 in Uganda (Ithete et al. 2013, Anthony et al. 2017a). While NeoCoV shares ∼85% nucleotide identity with human and camel MERS-CoV across the full genome, this drops to 64% in spike and 46% in S1 but similarity is 87.2% in S2 (Corman et al. 2014). This is analogous to the genetic similarity of PDF-2180 to MERS-CoV (Anthony et al. 2017a). This extensive dissimilarity in S1 explains the difference in host receptor usage. NeoCoV and PDF-2180 demonstrate ACE2 dependant cell entry, as shown using a pseudotyped virus model (Anthony et al. 2017a). Both these bat viruses have the capacity to bind several bat orthologues of ACE2, but with no notable affinity for hACE2. As was shown with BtkY72, a single T501F point mutation inserted into the RBD of NeoCoV resulted in higher efficiency entry into hACE2 cell lines; whereas the equivalent G501A in PDF-2180 only marginally increased hACE2 affinity. The close phylogenetic relationship of NeoCoV and PDF-2180 to MERS-CoV in the RdRp gene but dissimilarity in the S gene, is indicative of a recombination event in the origin of MERS-CoV which likely resulted in the capacity to bind human DPP4 (Corman et al. 2014). NeoCoV may pose a significant threat to human health due to the potential emergence of a hACE2 binding MERS-CoV-2 or human adapted NeoCoV (Anthony et al. 2017a).

The high fatality rate of MERS-CoV, diversity of merbecoviruses in bats, camels, and pangolins, and substantial genetic differences between known merbecoviruses and viruses against which population immunity has been generated, highlights a risk for future disease outbreak. While further research is needed to characterize the receptor usage and host range of the known catalogue of merbecoviruses, their presence across multiple animal reservoirs and potential for cross-species transmission suggest that they warrant continued attention in the context of pandemic preparedness.

## Protection against future pandemics

Performing large-scale surveillance of viral diversity in nature and developing reactionary therapeutics for each candidate virus is an unrealistic solution for preventing and/or mitigating the next pandemic. Instead, we must develop criteria to identify viruses of pandemic potential and prepare interventions that can target factors contributing to emergence and transmission. In the case of betacoronaviruses, measures to prevent emergence are likely to be behavioural, such as limiting contact between their natural bat reservoir, intermediate species and humans. On the other hand, prevention of transmission and limiting severity of disease can be achieved by pharmacological interventions such as vaccines, mAbs, and antivirals. While mAbs and antivirals are critical for a pandemic response, vaccines that prevent initial transmission to humans would be critical to prevent a future pandemic.

Neutralizing antibodies (nAbs) elicited from infection and/or vaccination prevent viral-host receptor engagement and entry into cells, and have been shown to correlate tightly with protection against infection and severe SARS-CoV-2 disease (Khoury et al. 2021, Khoury et al. 2023). The only currently licenced human coronavirus vaccines are for SARS-CoV-2; however, clinical trials have been conducted for SARS-CoV-1 and MERS-CoV vaccine candidates (Zhao et al. 2017). The majority of SARS-CoV-2 vaccines are based on the S protein of the virus, as the mediator of host cell entry and target of nAbs. However, as the most genetically variable region, the spike protein frequently acquires mutations that reduce the efficacy of these vaccines against SARS-CoV-2 variants (Cromer et al. 2022) and limits cross-reactivity with diverse mammalian betacoronaviruses. To prevent establishing vaccine escape by a novel, rapidly evolving virus, as seen with the SARS-CoV-2 variants, the development of a pan-coronavirus or pan-sarbecovirus vaccine has been proposed (Li et al. 2021, Nathan et al. 2021, Halfmann et al. 2022, Guo et al. 2023a, van Bergen et al. 2023, Bartsch et al. 2024, Cankat et al. 2024). Several strategies are currently being explored to achieve such broad protection including the design of novel chimeric spike antigens (Counoupas et al. 2025), and the use of nanoparticles displaying a range of betacoronavirus RBDs (Cohen et al. 2024). However, establishing a clear regulatory pathway for licensure of vaccines incorporating zoonotic virus antigens will be essential to expand their potential role in preventing future spillover events (Gordon et al. 2025). A 2024 modelling study evaluating the health and economic impact of a pan-coronavirus vaccine in the USA found that, even with just 10% efficacy against both infection and severe disease, such a vaccine could have prevented an estimated 14.6 million infections, 1.2 million hospitalizations, and 403 000 deaths during the initial SARS-CoV-2 outbreak (Bartsch et al. 2024). However, the rational development of a pan-coronavirus or pan-sarbecovirus vaccine would have to cover a very diverse population of potential mammalian viruses (Case et al. 2024, Lewitus et al. 2023). Assessing any cross-protection afforded by infection and/or vaccination with known coronaviruses may help to reveal gaps in coronavirus diversity that could be exploited in vaccine development.

## Cross-reactivity of SARS-CoV-2 induce nAb against diverse sarbecoviruses

Early in the SARS-CoV-1 and SARS-CoV-2 outbreaks, several studies assessed whether nAbs that had the capacity to recognize and neutralize these novel emergent viruses were present in the population. The viruses that were postulated to cross-react against SARS-CoV-1 were two of common cold coronaviruses known at the time–229E and OC43. Two hundred human serum samples were tested against SARS-CoV-1, and none were found to contain cross reactive nAbs (Jiang et al. 2024). In 2020, sera from patients who had been infected with SARS-CoV-1 were tested against live SARS-CoV-2. Again, nAbs conferred from SARS-CoV-1 infection did not neutralize SARS-CoV-2 (Anderson et al. 2020). This is not unexpected in the context of the substantial antigenic dissimilarity between these viruses; however, it highlights the potential for the current SARS-CoV-2 vaccine-induced nAbs present in the population to be likely ineffective against a future betacoronavirus outbreak. To this end, studies have begun to shed light on the cross-reactivity of SARS-CoV-2 vaccinated patient sera against novel mammalian betacoronaviruses. The initial publication describing WIV1 demonstrated that 7/9 sera samples from patients who had been infected with SARS-CoV-1 had the capacity to neutralize this novel bat virus (Ge et al. 2013). However, in a recent study using sera from SARS-CoV-2 mRNA vaccinated individuals, WIV1 showed a 44.3-fold reduction in neutralization compared to Ancestral SARS-CoV-2 (Garcia-Beltran et al. 2021).

The neutralization capacity of COVID-19 convalescent patient sera has similarly been tested against PCoV-GD and PCoV-GX. In line with the homology in the RBD to SARS-CoV-2, PCoV-GD was effectively neutralized by COVID-19 patient serum; however, neutralization of PCoV-GX, which is genetically more divergent, was considerably lower (Nie et al. 2021). Khosta-2, which is more distantly related to SARS-CoV-2 than the pangolin coronaviruses, is resistant to neutralization by sera from COVID-19 mRNA vaccinated individuals, as well as the SARS-CoV-2 mAb, Bamlinaviab (Seifert et al. 2022). Similarly, PRD-0038, which also belongs to clade 3, was poorly neutralized by a SARS-CoV-2 stabilized-spike vaccine candidate in a mouse model, compared to SARS-CoV-2 (D614G) and RaTG13 (Lee et al. 2023). NeoCoV is of particular interest in this respect, as it is able to bind ACE2 but is unrelated to SARS-CoV-1 and SARS-CoV-2. NeoCoV is resistant to neutralization by SARS-CoV-2 vaccinated human sera, and also largely resistant to MERS-CoV mAbs (Anthony et al. 2017a, Xiong et al. 2022). While not all circulating betacornaviruses have been assessed for cross-neutralization by sera from SARS-CoV-2 infected and/or vaccinated individuals, the available data suggest that immunity induced by SARS-CoV-2 infection or vaccination may not provide broad protection against genetically divergent betacoronaviruses.

## Antivirals

Antivirals, such as Remdesivir, Molnupiravir, Paxlovid, and Ensitrelvir were approved for use against SARS-CoV-2 infection. Broad spectrum antivirals, such as Remdesivir and Molnupiravir, frequently target conserved viral enzymes, which act as nucleoside analogues (Sheahan et al. 2020, Simonis et al. 2021). As catalytic motifs of RNA polymerases are highly conserved within and between viral families, RdRp inhibitors can be effective against a wide range of viral families. Several of these were repurposed for prophylactic and therapeutic use in the SARS-CoV-2 pandemic (Artese et al. 2020, Simonis et al. 2021, Iketani and Ho 2024). For example, Molnupiravir has shown efficacy against influenza A/B virus, Ebola virus, Chikungunya virus, MERS-CoV, and a range of SARS-CoV-2 variants (Sheahan et al. 2020). The accelerated development of Nirmatrelvir, the antiviral component of Paxlovid, was made possible by earlier drug discovery efforts targeting SARS-CoV-1. Both Nirmatrelvir and Ensitrelvir inhibit the main viral protease (3CL^pro^), a target that is highly conserved across alpha- and betacoronaviruses (Zhang et al. 2020b, Unoh et al. 2022). Given their broad antiviral activity across genera and families, these compounds may have an important role in responding to future coronavirus pandemics. However, as with mAbs, the emergence of antiviral resistance mutations during large-scale or uncontrolled outbreaks may limit their long-term effectiveness (Tamura et al. 2024, Zhang et al. 2024).

## Future directions and conclusions

Human encroachment on previously undisturbed animal habitats, the rise of the wildlife trade, as well as the impact of climate change, are increasing the frequency of zoonotic spillover events. Modelling predicts the yearly probability of extreme epidemics will increase three-fold in the next few decades (Marani et al. 2021). As exemplified by the COVID-19 pandemic, the introduction of a new pathogen into a population with little-to-no pre-existing immunity can result in outbreaks with high morbidity and mortality and have devastating economic impacts. Antigenically diverse betacoronaviruses with pandemic potential are circulating in nature and it is inevitable that further spillover of these viruses will occur. Coinfection of bats by these viruses enables the ongoing emergence of novel viruses with unique genotypic combinations via recombination. Subsequently, surveillance alone will be insufficient in determining the pandemic risk posed by these viruses (Wells et al. 2023). Proactive measures, such as the development of pan-coronavirus vaccine libraries, broad-spectrum mAbs, and new antivirals may offer effective risk-mitigation strategies and should be prioritized as part of comprehensive future pandemic preparedness efforts.
